# Phenol–Formaldehyde Adhesives Modified with Eucalyptus Lignin: The Advantages of Soda Lignin

**DOI:** 10.3390/polym17243319

**Published:** 2025-12-16

**Authors:** Leonardo Clavijo, Rodrigo Coniglio, Fabián Bermúdez, Juan Martín Rodao, Diego Passarella, Andrés Dieste

**Affiliations:** 1 Chemical Engineering Institute, Faculty of Engineering, Universidad de la República, Julio Herrera y Reissig 565, Montevideo 11300, Uruguay; rconiglio@fing.edu.uy (R.C.); fbermudez@fing.edu.uy (F.B.); jmrodao@fing.edu.uy (J.M.R.); 2 Regional University Center of the Northeast, Universidad de la República, Route 5, km. 386.5, Tacuarembó 45000, Uruguay; diego.passarella@noreste.udelar.edu.uy; 3 Independent Consultant, 27002 Lugo, Spain; andres.dieste@ejemadera.com

**Keywords:** lignin, Eucalyptus, extender, activation, adhesives

## Abstract

This study investigates the performance of phenol–formaldehyde adhesives containing Eucalyptus lignin as an extender in their formulation. A commercial phenol–formaldehyde resin was used, and five different types of lignin were tested: (1) kraft lignin precipitated with carbon dioxide, (2) kraft lignin precipitated with sulfuric acid, (3) soda lignin precipitated with hydrochloric acid, (4) soda lignin precipitated with sulfuric acid, and (5) a second soda lignin where the wood underwent a phosphoric acid extraction process prior to alkaline extraction. The lignins were used both unmodified and activated through three different processes: hydroxymethylation, phenolysis in an acidic medium, and alkaline phenolysis. Adhesives were formulated with substitution percentages of the base resin ranging from 10% to 60%, in addition to a reference adhesive that contained no lignin. Wooden test specimens were manufactured to determine the tensile shear strength. Results indicate that best performance is achieved when lignins are activated through hydroxymethylation and when soda lignin is used. Under optimal conditions, it is possible to replace at least 45% of the base resin with activated Eucalyptus soda lignin, which represents a reduction of at least 30% in the cost of the final adhesive. This substitution results in a 46% increase in adhesive strength compared to the base adhesive (without lignin). These findings suggest that the valorization Eucalyptus soda lignin could have significant economic and environmental benefits.

## 1. Introduction

Adhesives play a critical role across many industries, and their market is continuously growing, totaling approximately USD 69 billion in 2024. Most adhesives are produced from petroleum derivatives, which also have an increasing demand, impacting their cost. This fact, combined with heightened environmental awareness, has led to extensive research into the formulation of adhesives using sustainable components [1,2,3,4]. In the wood processing industry, phenol–formaldehyde resin-based adhesives represent over 90% of the adhesives consumed, with a market of USD 4 billion in 2024, having shown a steady growth of 5% since 2019 [4,5]. Partial replacement of phenol with lignin can contribute to reducing the final adhesive cost and achieving a formulation with lower dependency on petroleum-derived products.

Phenol-formaldehyde resin, commercially introduced in the 1930s after its discovery in the late 19th century, is widely used in the manufacture of plywood, laminated products, and other engineered wood composites. Its key advantages include high moisture resistance, strong cohesion, excellent dimensional stability, and relatively low cost. These resins are suitable for a variety of wood bonding applications, provided the adhesive joints can be heat-cured [6,7,8].

Ready-to-use (RTU) adhesives are formulated for immediate application and comprise multiple components that determine their final properties. These components include:

- Base resin: the active component responsible for adhesion, and from which the adhesive derives its name.
- Solvent: usually water, which is released during adhesive curing
- Curing agents or hardeners: chemicals that react with the base resin to induce polymerization and are added at the time of adhesive application
- Fillers: inert solids added to reduce the amount of base resin and lower the final adhesive cost. Generally, they do not exhibit adhesive behavior by themselves, but can decrease adhesive penetration into the wood structure or can increase the final viscosity.
- Extenders: substances with limited adhesive properties are also added to partially replace the base resin. Wheat flour is a common extender in phenol–formaldehyde adhesive formulations.
- Stabilizers are chemicals added to enhance adhesive durability under specific conditions.
- Diluent: a liquid added to reduce viscosity and facilitate application.
- Catalysts or accelerators: agents added in very small amounts that speed polymerization during curing.
- Additives: compounds imparting specific properties such as flame retardancy, UV stability, or coloration.

Lignin, the most abundant renewable source of aromatic compounds, structurally resembles phenol due to its phenylpropane units and contains reactive groups such as phenolic and aliphatic hydroxyls, carbonyls, and carboxyls capable of reacting with formaldehyde and phenol during resin synthesis [9]. This positions lignin as a potential phenol substitute in adhesive formulations. However, its complex structure, steric hindrance, and fewer reactive aromatic sites—estimated at only 0.3 per C9 lignin unit, about one-tenth that of phenol—have limited its practical application in adhesives [10].

To overcome these limitations, chemical modification or “activation” of lignin has been proposed to increase its reactive sites or introduce new functional groups [2,9,10,11,12,13,14]. Such modifications enable phenol substitution levels of 30–50% in phenol–formaldehyde resins, yielding adhesives with acceptable physical and mechanical properties, although adhesion strength typically remains lower than that of commercial controls [15]. In some cases, it was also necessary to increase the pressing time required for adhesive curing.

The main methods used to activate lignin are hydroxymethylation, oxidation, demethylation, and phenol modification (phenolysis).

- The goal of hydroxymethylation is to increase reactive sites by introducing hydroxymethyl groups (-CH_2_OH) into lignin molecules through electrophilic aromatic substitution when lignin is mixed with formaldehyde in an alkaline medium between 75 °C and 90 °C. This method does not increase the number of active sites, but significantly increases lignin reactivity [2,4,9].
- The oxidation of lignin causes its crosslinking, and is achieved by (1) reacting the phenolic ring using H_2_O_2_ in the presence of SO_2_; or by (2) using enzymes to activate polymerization [16]
- Demethylated lignin is obtained by reacting it with a strong oxidizing agent, like K_2_Cr_2_O_7_, forming catechol groups, decreasing the concentration of methoxyl groups and increasing that of phenolic hydroxyl groups [17].
- Phenolic modification is one of the most used methods for lignin activation [9]. During this process, phenol molecules are incorporated into the lignin structure, but ether linkages in the lignin molecule are also broken, reducing its molecular weight and polydispersity. The process can be carried out in either acidic or basic media. While acidic phenolysis is the most used procedure, basic phenolysis has the advantage that, occurring in the pH range of adhesive synthesis, the mixture obtained after phenolysis can be directly incorporated into the adhesive formulation [18,19].

In this study, hydroxymethylation is selected as one of the activation methods, as it has previously shown promising results when applied to Eucalyptus lignin for other uses [20]. Additionally, both acid and alkaline phenolysis are considered, as these techniques have demonstrated the most favorable outcomes in the preparation of lignin–phenol–formaldehyde resins.

There are two alternatives for incorporating lignin in adhesive manufacturing: it can be a component of the resin, included as a reactant during its production, or the resin can be used as is, with lignin serving as an extender when the RTU adhesive is prepared [21]. Although most of the research follows the first approach and better results have been reported when lignin is included in the resin formulation, phenol–formaldehyde resin is not produced in many countries but imported. Therefore, using lignin as an extender could be a solution in such cases, as the aim in adhesive formulation is to reduce the amount of base resin used, which is the most expensive component.

The difference between the two methods of incorporating lignin into the final adhesive is substantial. In the first case, lignin must be incorporated as a reactant in the resin production plant, where it must polymerize and crosslink in the same way that phenol does with formaldehyde. For this process to be successful, lignin must closely resemble phenol; lignins with low molecular weight and minimally substituted aromatic rings are those most likely to succeed. Additionally, the resin formation process is complex, requiring controlled temperature and pressure conditions. In the case of using lignin as an extender, it is mixed with the PF resin at the engineered wood product (EWP) production plant, just before the adhesive is applied. Here, the process is much simpler, as the lignin is added as just another ingredient during the mixing stage of the ready-to-use (RTU) adhesive. In this case, the best results are achieved when the lignin size is similar to that of the partially crosslinked resin, as the curing process is more efficient, leading to a more stable and homogeneous polymer network [22,23].

In Uruguay and much of Latin America, *Eucalyptus* species dominate the wood industry, serving as the primary source for both pulp production and the manufacture of boards and panels. Consequently, the valorization of by-products generated by these industries represents a promising area of research that warrants further investigation. *Eucalyptus* lignin is distinguished by its relatively low reactivity compared to other types of lignin, primarily due to its high syringyl/guaiacyl (S/G) ratio. As a result, findings derived from studies on coniferous lignins may not be directly applicable to *Eucalyptus* lignin. The aim of this study is to develop adhesive formulations incorporating varying proportions of unmodified and chemically activated kraft and soda *Eucalyptus* lignins as extenders, partially replacing phenol–formaldehyde (PF) resin, and to assess the resulting adhesive properties. Specifically, the objectives are to determine the necessity of activating *Eucalyptus* lignin, identify the activation method that yields optimal performance, and establish the maximum feasible substitution level of PF resin with lignin.

## 2. Materials and Methods

### 2.1. Raw Material

Five different lignins from *Eucalyptus* wood were used. Two kraft lignins were obtained by acid precipitation from industrial black liquor provided by UPM-Fray Bentos (Uruguay), a mill that processes a mix of woods including *Eucalyptus grandis*, *Eucalyptus dunnii*, *Eucalyptus maidenii*, and *Eucalyptus globulus*. One of these lignins (KL1) was precipitated with carbon dioxide, and the other was precipitated with sulfuric acid (KL2) at pH = 9. The precipitation was conducted in a pilot plant according to the procedure described in [24]. Additionally, a lignin obtained by acid precipitation of black liquor from a soda treatment of *Eucalyptus* sawdust from the same mill was used, as described in [25]. For precipitation, both hydrochloric acid (SL1) and sulfuric acid (SL2) were used. A fifth lignin obtained by acid precipitation of black liquor from a soda treatment of *Eucalyptus* sawdust previously subjected to phosphoric acid treatment to recover xylose and acetic acid, as described in [26], was also used. In this case, precipitation was carried out with sulfuric acid at pH = 9 and lignin SL3 was obtained.

The starting lignins were characterized by determining the content of phenolic hydroxyl (-OH) groups, the S/G ratio, and the molecular weight distribution. The determination of phenolic hydroxyl group content was performed according to the procedure with the reagent of Folin and Ciocalteu, compiled in [27]. The S/G ratio was determined by nitrobenzene oxidation by a modification of the Lin and Dence procedure [28]. Briefly, approximately 0.05 g of oven-dried lignin sample was treated with 7 mL of 2 M NaOH aqueous solution and 0.5 mL nitrobenzene in glass reactors and heated up to 170 °C for 2.5 h. The oxidation products were then extracted with 30 mL of chloroform. The aqueous phase is then transferred to a centrifuge tube, and 2.5 mL of 4 M HCl is added. The sample is centrifuged, the liquid filtered and placed in an HPLC vial. The solutions were analyzed by an HPLC system (Shimadzu, Kyoto, Japan) equipped with a Purospher Star RP-18 column, with acetonitrile/water (1:6, *v*/*v*) as the eluent. The column temperature was maintained at 40 °C, and an eluent flow rate of 1.0 mL/min was used. Vanillin and vanillic acid, and syringaldehyde and syringic acid standards were used for the quantification of guaiacyl and syringyl unit derivatives, respectively.

The molecular weight distribution was determined by gel permeation chromatography (GPC), in an alkaline medium, according to procedure ILI008, as reported in [29]. As a stationary phase, 5 columns PSS MCX of 1000 Å, 8 × 300 mm, 10 µm were used, which separate anionic polymers in the range of 200 to 70,000 Da. The column temperature was maintained at 35 °C, and an eluent flow rate of 0.6 mL/min was used. Sodium salts of polystyrene sulfonate from PSS (Polymer Standards Service) with weighted molecular weights of 890, 2000, 3200, 4200, 6000, 10,000, 15,000, 20,000, 30,000 and 67,000 Da were used as standards, which were dissolved in the mobile phase at a concentration of 1 g/L for 48 h.

### 2.2. Lignin Activation

To activate the precipitated lignin, hydroxymethylation (HM) with formaldehyde, acidic phenolysis (AP), and basic phenolysis (BP) were chosen as procedures. The hydroxymethylation procedure involves dissolving lignin in soda with a m_NaOH_/m_Lignin_ ratio of 0.17 and then treating it with formaldehyde with a m_CH2O_/m_Lignin_ ratio of 0.333 at 45 °C for 5 h under reflux with magnetic stirring. These conditions maximize hydroxymethyl groups in lignin and minimize Cannizzaro side reactions and free formaldehyde. A solution with a 27% (m/m) lignin concentration is obtained. For acidic phenolysis, the technique described in [18] is followed. In a three-necked flask, a mixture of lignin and phenol is prepared with an m_Phenol_/m_Lignin_ ratio of 1.67 and a 5% concentration of sulfuric acid. The flask is heated to 90 °C with reflux and magnetic stirring for 2 h. The flask is then cooled, and 20 mL of ethyl acetate is added to dissolve the mixture, which is transferred to a beaker. Subsequently, 100 mL of petroleum ether is added to precipitate the suspension, which is filtered, washed, and dried in an oven. For basic phenolysis, the technique described in [19] is followed. In a three-necked flask, an equal amount of lignin and phenol is added, and the mixture is dissolved in 30% soda, maintaining the pH between 9 and 10. The mixture is heated to 95 °C and maintained at this temperature for 1 h with magnetic stirring and reflux. It is then cooled and transferred to a beaker, where a 1:1 (*v*/*v*) diethyl ether/water solution is added. The pH of the mixture is adjusted to 2 with the same acid used for lignin precipitation. The precipitated lignin is centrifuged, then washed with diethyl ether, and dried in an oven.

### 2.3. Adhesive Manufacture

To fabricate the adhesives, a commercial phenol–formaldehyde resin provided by Hexion (Tacuarembó, Uruguay) was used, with a nominal solids content of 43%, a viscosity of 800 ± 300 cP, a pH of 11.5 ± 0.5, and a density of 1.19 ± 0.01 g/cm^3^. The solids content was determined before adhesive fabrication, and the formulation was adjusted according to the obtained value. Walnut shell flour was used as a filler, and wheat flour as an extender, both provided by Hexion. A base adhesive (which does not contain lignin) was fabricated to serve as a control.

Formulations for all different adhesives are presented in Table 1 and Table 2. The formulation for the base adhesive was suggested by Hexion and is based on [30]. To prepare the adhesive, the wheat flour was dispersed in water and mixed for 2 min. Then, the walnut shell flour was added and mixed for 5 min, ensuring that no lumps formed. A portion of the commercial resin (approximately one-third) was added, followed by sodium hydroxide. The mixture was stirred for 20 min. Finally, the remaining resin was added and stirred for an additional 5 min. To prepare the adhesive with activated lignin as an extender, in the case of hydroxymethylated lignins, it is necessary to reduce the amounts of soda and water added, as these are present in the modified lignin solutions. The lignin is added to the system after the sodium hydroxide solution. In the case of unmodified lignin and lignins modified by acidic and basic phenolysis, as they are solids, they are added during adhesive fabrication. In all cases, the lignin substitution percentages are calculated based on the dry solids content of the phenol–formaldehyde resin.

The viscosity of the obtained adhesive was controlled using a Brookfield DV2T viscometer (Toronto, ON, Canada).

### 2.4. Fabrication of Test Pieces and Determination of Tensile Shear Strength

Test pieces were fabricated according to the European standard EN 205:2016 [31], from knot-free pine wood cut along the wood grain, measuring 150 mm in length, 20 mm in width, and 5 mm in thickness. The adhesive was applied to both pieces of wood at a rate of (227 ± 10) g/m^2^, and the pressing was carried out in a Chengyi CHY-600DG hot press (Zhengzhou, China) at a temperature of 130 °C and a pressure of 10 bar for 10 min. After pressing, the specimens were allowed to cool and are stored. After 5 days, two cuts were made, 10 mm apart, and the specimens were conditioned in a controlled atmosphere at (20 ± 2) °C and a relative humidity of (65 ± 5)% for 7 days. The maximum tensile strength was then determined using a universal testing machine at a rupture speed of 6 mm/min.

## 3. Results

### 3.1. Lignin Characterization

In Table 3, the results of starting lignin characterization are presented.

The main differences between the various types of lignin are attributed to the higher content of phenolic OH groups in kraft lignin compared to soda lignin, and the larger size of soda lignin relative to kraft lignin, with the weight average molecular weight (Mw) of soda lignin being more than twice that of kraft lignin. The S/G ratio for all lignins shows the same value within the experimental error, indicating that syringyl structures account for 75% of the aromatic structures, while guaiacyl structures make up the remaining 25%.

### 3.2. Lignin Activation

**Figure 1:**
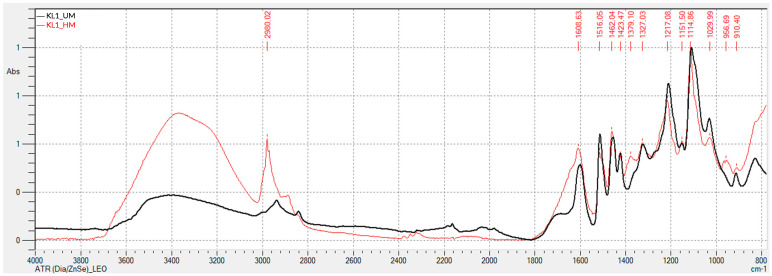
FTIR-ATR Spectrum of Unmodified and Hydroxymethylated Kraft Lignin Precipitated with CO_2_.

**Figure 2:**
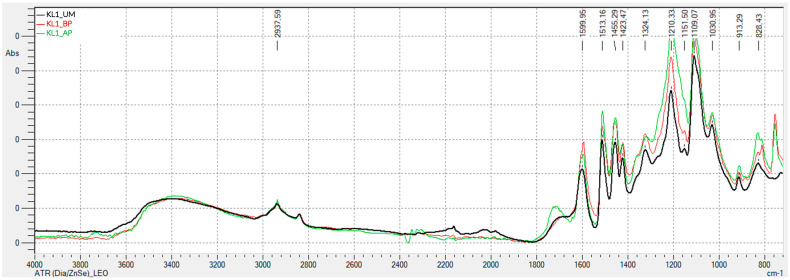
FTIR-ATR Spectrum of Unmodified and Phenolysis-Modified Kraft Lignin Precipitated with CO_2_.

Figure 1 and Figure 2 present the FTIR-ATR spectra obtained for kraft lignins precipitated with CO_2_: unmodified (KL1_UM), hydroxymethylated (KL1_HM), modified with basic phenolysis (KL1_BP), and modified with acid phenolysis (KL1_AP) lignins.

Comparing the spectra of the unmodified lignin with the hydroxymethylated lignin reveals that the latter exhibits a more prominent band around 3350 cm^−1^, corresponding to aromatic and aliphatic hydroxyl (-OH) groups. The peak at 2980 cm^−1^, attributed to the stretching of -CH_2_ and -CH_3_ groups, is also more intense in the hydroxymethylated lignin. The peak at 1462 cm^−1^, associated with the asymmetric bending of -CH_2_ and -CH_3_ groups, is more pronounced for the hydroxymethylated lignin as well. The peak at 1030 cm^−1^, corresponding to the guaiacyl structure vibration, decreases in intensity for the hydroxymethylated lignin. These changes suggest a greater presence of hydroxymethyl groups in the lignin structure, consistent with the activation process performed.

Analyzing the FTIR-ATR spectra of kraft lignin precipitated with CO_2_, which was subsequently activated through acid and basic phenolysis, shows that the band around 3400 cm^−1^, corresponding to hydroxyl groups, is similar for all three lignins. The peak at 1708 cm^−1^, assigned to the C=O vibration in carbonyl groups not conjugated with the aromatic ring, is only present in the acid-phenol modified lignin. Peaks at 1324 cm^−1^, 1210 cm^−1^, and 1109 cm^−1^ are attributed to the vibration of phenolic -OH groups, C-C and C-O vibrations in guaiacyl units, and aromatic C-H deformation in syringyl units, respectively, and are more intense in phenolated lignins. Peaks at 1151 cm^−1^ and 1031 cm^−1^ correspond to the vibration of secondary and primary aliphatic -OH groups, respectively, and are more intense in phenolated lignins. In this case, the observed changes are in the intensities of peaks and bands, as the phenolysis process introduces phenol groups, which exhibit similar bands to lignin.

**Figure 3:**
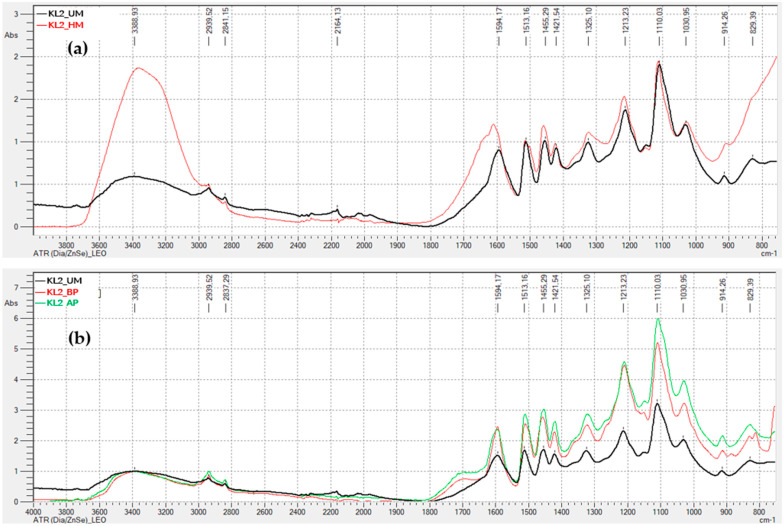

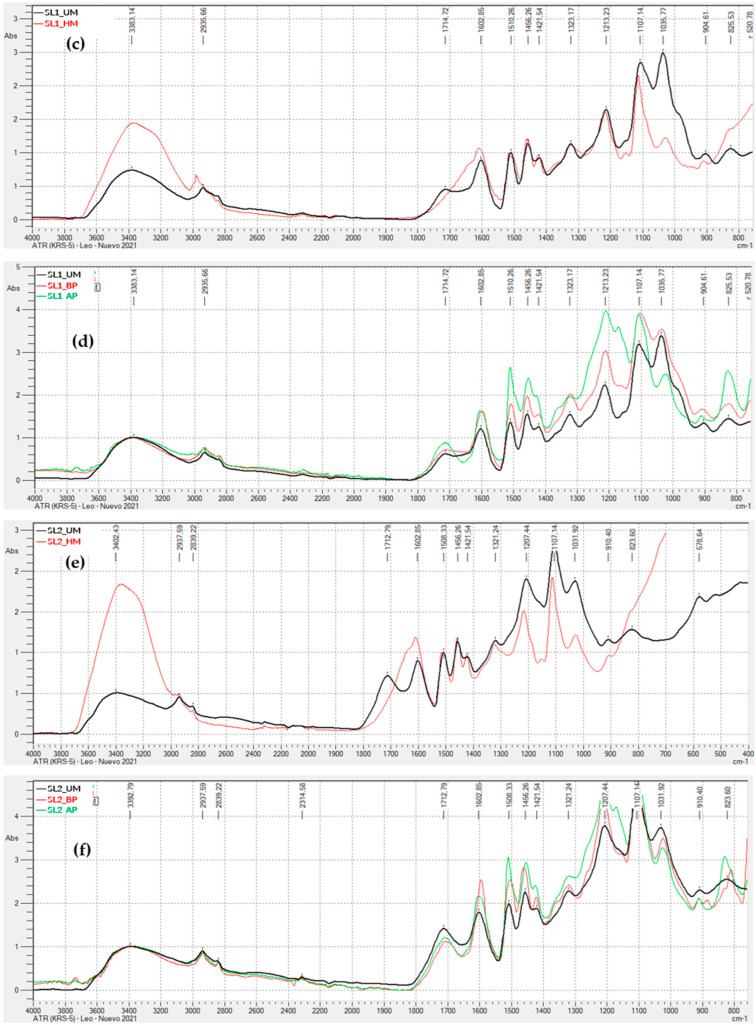

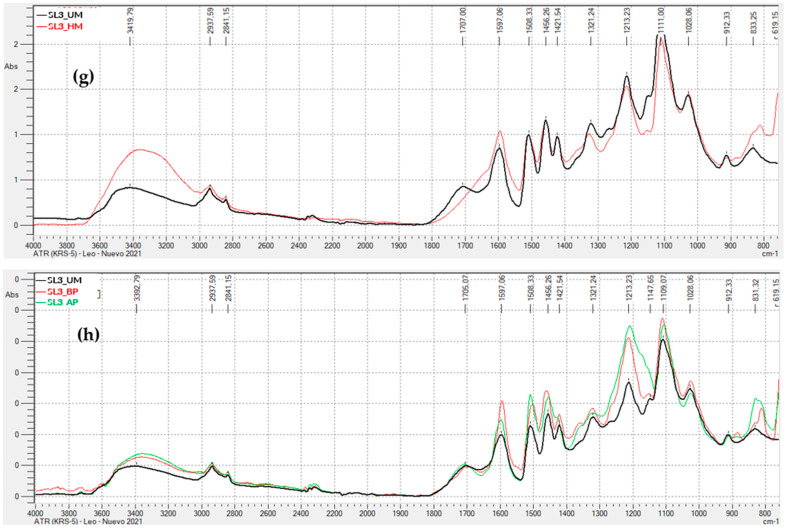
FTIR-ATR Spectra of Unmodified and Modified Lignins: (**a**) Kraft lignin precipitated with H_2_SO_4_ (KL2), hydroxymethylated (HM) and unmodified (UM); (**b**) Kraft lignin precipitated with H_2_SO_4_ (KL2), modified by acid phenolysis (AP), basic phenolysis (BP), and unmodified (UM); (**c**) soda lignin precipitated with HCl (SL1), hydroxymethylated (HM) and unmodified (UM); (**d**) soda lignin precipitated with HCl (SL1), modified by acid phenolysis (AP), basic phenolysis (BP), and unmodified (UM); (**e**) Soda lignin precipitated with H_2_SO_4_ (SL2), hydroxymethylated (HM) and unmodified (UM); (**f**) Soda lignin precipitated with H_2_SO_4_ (SL2), modified by acid phenolysis (AP), basic phenolysis (BP), and unmodified (UM); (**g**) Soda lignin 3 precipitated with H_2_SO_4_ (SL3), hydroxymethylated (HM) and unmodified (UM); (**h**) Soda lignin 3 precipitated with H_2_SO_4_ (SL3), modified by acid phenolysis (AP), basic phenolysis (BP), and unmodified (UM).

Figure 3 display the FTIR-ATR spectra for the remaining lignins (kraft precipitated with sulfuric acid, and soda lignins precipitated with hydrochloric acid and sulfuric acid), both unmodified, hydroxymethylated, and modified through acid and basic phenolysis. Generally, the behavior is similar to that observed for the kraft lignin precipitated with CO2, indicating that the lignin modification treatments were successful.

In summary, the ATR-FTIR spectra of the activated lignins exhibit changes in the observed peaks that are consistent with the chemical modifications occurring during activation, for both hydroxymethylation and acid and alkaline phenolysis.

### 3.3. Evaluation of Adhesives

Table 4, Table 5, Table 6 and Table 7 present the statistical analysis of the tensile strength obtained for the base adhesive and the adhesives containing each of the tested lignins, conducted using an ANOVA test with Fisher’s Least Significant Difference (LSD) procedure, at a 95% confidence level. The mean strength, standard error, and results of group comparisons are indicated. Groups sharing a common letter have no statistically significant differences, whereas groups with different letters exhibit significant differences. The final column indicates whether there is a significant difference from the base adhesive. Prior to performing the ANOVA tests, normality and homogeneity of variances were assessed using the Shapiro–Wilk test and Levene’s test, respectively.

**Figure 4:**
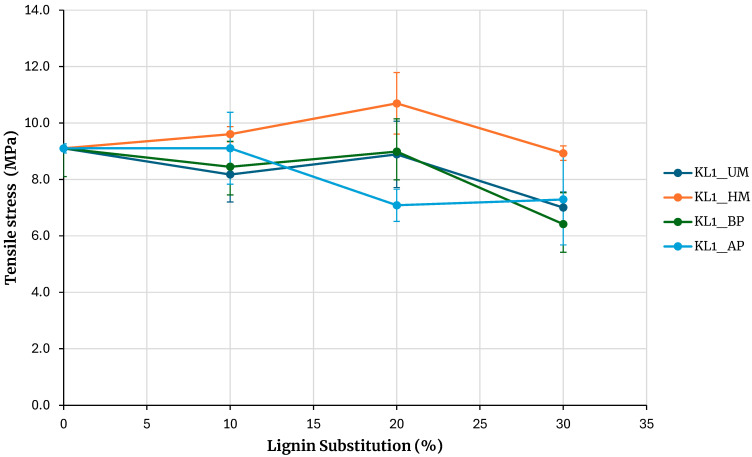
Evaluation of adhesives manufactured with kraft lignin precipitated with CO_2_.

**Figure 5:**
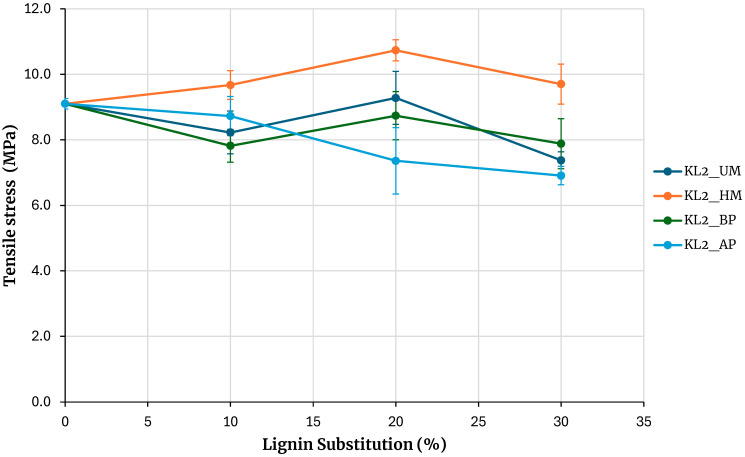
Evaluation of adhesives manufactured with kraft lignin precipitated with H_2_SO_4_.

**Figure 6:**
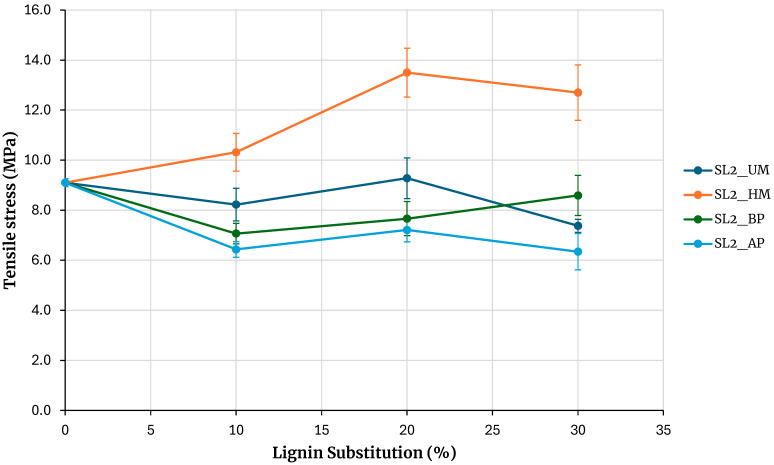
Evaluation of adhesives manufactured with soda lignin precipitated with H_2_SO_4_.

**Figure 7:**
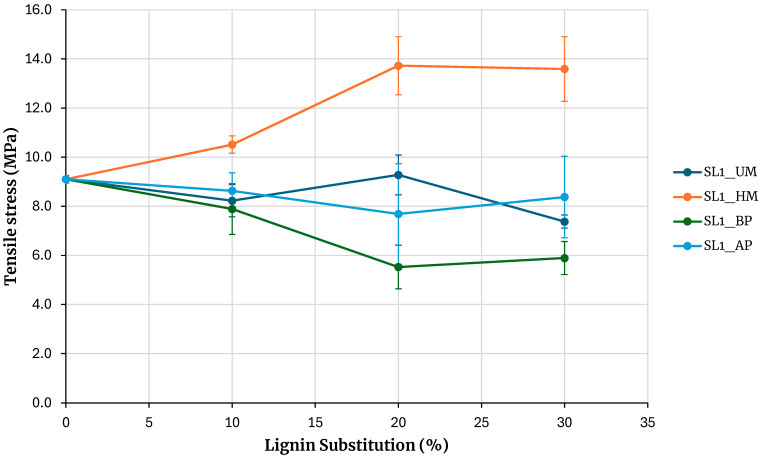
Evaluation of adhesives manufactured with soda lignin precipitated with HCl.

**Figure 8:**
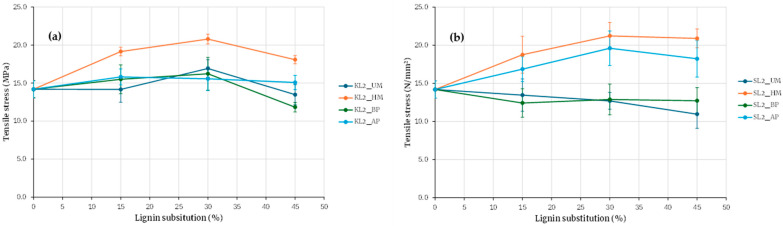
Evaluation of adhesives manufactured with kraft lignin (**a**) and soda lignin (**b**) precipitated with H_2_SO_4_ with higher lignin substitution.

**Figure 9:**
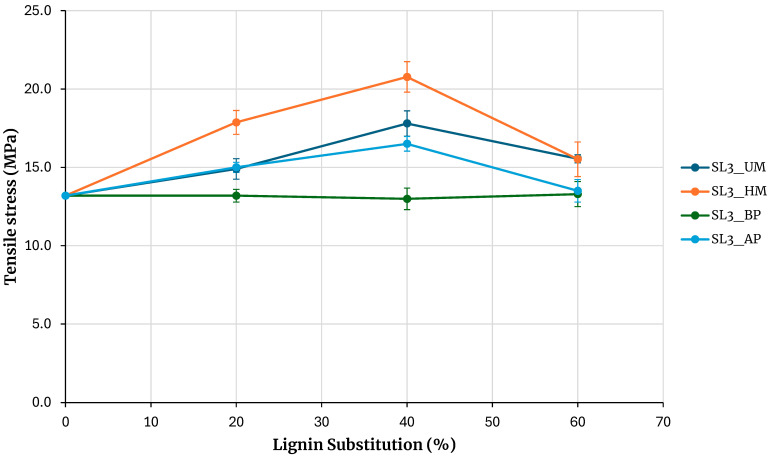
Evaluation of adhesives manufactured with soda lignin 3 with higher lignin substitution.

**Figure 10:**
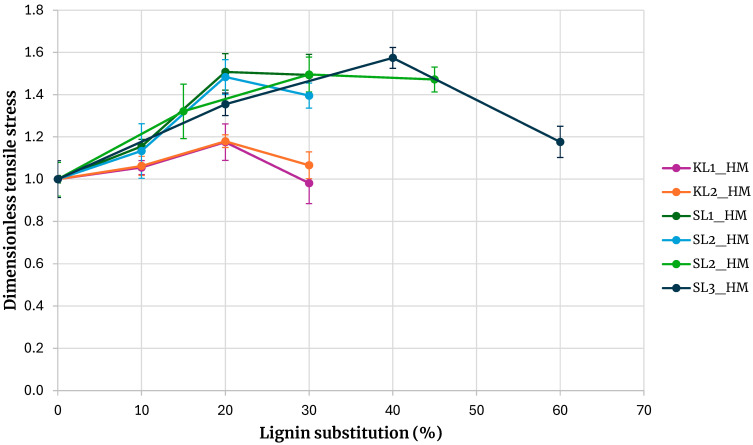
Comparison of adhesives with hydroxymethylated lignin by origin.

Figure 4 shows the maximum tensile strengths obtained from testing samples of adhesives based on kraft lignin precipitated with CO_2_, including unmodified lignin (KL1_UM), lignin activated by hydroxymethylation (KL1_HM), acid phenolysis (KL1_AP), and basic phenolysis (KL1_BP), with substitution percentages of 10%, 20%, and 30%. The reference adhesive was prepared without lignin. The error bars in Figure 4 to 10 correspond to the standard error, calculated as the sample standard deviation over the square root of the sample size.

Increasing the substitution percentage of phenolic resin with lignin tends to decrease the adhesive’s bond strength for both unmodified lignin and lignin modified with acid and basic phenolysis treatments. For a 30% substitution percentage, the strength is lower than that of the base adhesive for both unmodified and phenolated lignin, although the decrease is not significant. An exception is the hydroxymethylated lignin, where the maximum rupture strength increases for 10% and 20% substitution percentages and matches the base adhesive strength at a 30% substitution percentage.

Figure 5 presents the maximum tensile stresses obtained from testing the specimens for adhesives prepared with kraft lignin precipitated with sulfuric acid. The reference is the adhesive prepared without lignin. Increasing the percentage of phenolic resin substitution with lignin results in a decrease in the adhesive’s strength for both unmodified lignin and lignin modified by acidic and basic phenolysis treatments. For a 30% substitution, the obtained strength is lower than that of the base adhesive, although not significantly. An exception to this behavior is observed with hydroxymethylated lignin, where the strength increases with the percentage of substitution, and at a 30% substitution, the maximum rupture stress obtained is higher than that of the base adhesive.

Figure 6 shows the maximum tensile stresses obtained from testing specimens for adhesives prepared with soda lignin precipitated with sulfuric acid. The reference is the adhesive prepared without lignin. Increasing the percentage of phenolic resin substitution with lignin maintains the strength for unmodified lignin and lignin modified with basic phenolysis, and tends to decrease for lignin modified with acidic phenolysis. At a 30% substitution, the strength is similar to that of the base adhesive when using lignin modified with basic phenolysis and slightly lower when using unmodified lignin and lignin modified with acidic phenolysis. An exception to this behavior is observed with hydroxymethylated lignin, where the strength increases with the percentage of substitution, and at a 30% substitution, the maximum rupture stress obtained is higher than that of the base adhesive. Although the behavior is similar to that of kraft lignin precipitated with sulfuric acid, the strength increase for a 30% substitution in kraft lignin is 6.6%, while in this case, it is 40%.

Figure 7 shows the maximum tensile stress obtained from testing specimens for adhesives prepared with soda lignin precipitated with hydrochloric acid. The reference is the adhesive prepared without lignin. Increasing the percentage of phenolic resin substitution with lignin maintains the strength for unmodified lignin and for lignin modified with acidic phenolysis. In the case of lignin modification through alkaline phenolic reaction, the resulting adhesive strength is significantly lower than that of the unmodified lignin across all tested substitution levels. This contrasts with kraft lignin, where the strength of the adhesive modified by alkaline phenolic reaction is comparable to that of the unmodified lignin, within the experimental error limits, thereby contradicting the intended objective. These findings suggest that alkaline phenolic reaction is not an effective method for activating lignin when used as an extender in adhesive formulations.

An exception to this behavior is observed with hydroxymethylated lignin, where the strength increases with the percentage of substitution, and at a 30% substitution, the maximum rupture stress obtained is higher than that of the base adhesive. Although the behavior is similar to that of kraft lignin precipitated with sulfuric acid, the strength increase for a 30% substitution in kraft lignin is 6.6%, whereas in this case, it is 49%. A 7% increase in stress is also observed when compared with lignin precipitated with sulfuric acid, although this increase falls within the error margin of the measurement.

Given the higher tensile strength achieved with hydroxymethylated lignin, adhesives with increased lignin substitution levels were subsequently formulated. Substitution ratios of 15, 30, and 45% were employed for Kraft and soda lignins precipitated with sulfuric acid, while ratios of 20, 40, and 60% were used for SL3 lignin. The results, presented in Figure 8 and Figure 9, are consistent with previous findings and further underscore the superior performance of hydroxymethylated lignins—particularly the enhanced tensile strength obtained with hydroxymethylated soda lignin.

For the adhesive made from hydroxymethylated kraft lignin (Figure 8a), an increase in strength is observed with the substitution percentage; the strength obtained with 30% substitution is 47% higher than that of the base adhesive, and with 45% substitution, it is 27% higher. Although a drop in strength is observed for a 45% replacement, it is still higher than that of the base adhesive, which suggests that it is feasible to formulate an adhesive with hydroxymethylated kraft lignin at a 45% substitution percentage and that further increases in substitution percentage may be possible.

Regarding the adhesive prepared with soda lignin (Figure 8b), for a 30% substitution percentage, the strength is 50% higher than that of the base adhesive, and for a 45% substitution percentage, it is 46% higher. In this case, there is no such pronounced drop in resistance when the substitution percentage is increased from 30 to 45% for soda lignin, in contrast to what happens with kraft lignin. The difference in this behavior could be explained by the size difference between kraft lignin and soda lignin, the latter being considerably larger, as shown in Table 3, which allows for the formation of a more stable polymer network, and consequently better adhesion [22,23]. When comparing results based on the lignin origin, it is observed that better maximum strength values are obtained with soda lignin compared to kraft lignin at the maximum substitution percentage tested. The results suggest that higher substitution percentages can be achieved with soda lignin compared to kraft lignin, potentially exceeding 45%.

Regarding the adhesive prepared with SL3, although there is a reduction in tensile strength at a 60% substitution level compared with the 45% substitution condition, the strength values at this highest level remain comparable to those of the unmodified PF adhesive.

Table 8 presents the analysis of variance for the results obtained with the new percentages tested. The table includes the average breaking strength, the standard error, and the results of the multiple comparisons to determine which means are significantly different from others. Eight homogeneous groups were identified, with no statistically significant differences among groups sharing at least one letter. The last column of the table indicates whether there is a significant difference compared to the base adhesive. The results indicate that adhesives with significant differences compared to the reference adhesive include those with kraft lignin hydroxymethylated at all three substitution percentages tested, soda lignin at all three substitution percentages tested, and soda lignin with acid phenolysis treatment at substitution percentages of 30% and 45%.

Figure 10 shows the tensile strength of adhesives formulated with hydroxymethylated lignins from different sources. For comparative purposes, all values were normalized by dividing them by the tensile strength of the base adhesive. A clear distinction is evident between adhesives prepared with kraft lignin and those prepared with soda lignin.

Kraft lignins precipitated with carbon dioxide or sulfuric acid exhibit similar behavior, as do soda lignins precipitated with hydrochloric or sulfuric acid. Across all substitution levels, adhesives containing kraft lignin show lower tensile strength than those containing soda lignin. At a 30% substitution level, the maximum tensile strength obtained with soda lignins is, on average, 40% higher than that obtained with kraft lignins. These findings indicate that adhesives incorporating soda lignin can tolerate higher substitution levels than those incorporating kraft lignin, with substitution ratios of at least 45% remaining feasible.

In summary, the evaluation of all adhesive formulations indicates that lignin activation through hydroxymethylation is essential for achieving higher levels of PF resin substitution with lignin. Substitution of up to 60% of the PF resin with lignin is feasible, resulting in an adhesive with greater strength than the baseline formulation; however, the optimal strength is observed at a 45% substitution level. A comparison between kraft and soda lignins reveals that soda lignin yields superior performance, likely due to its larger molecular size. When the resin (which is pre-crosslinked) and the extender have similar molecular weights, the adhesive curing process becomes more efficient, promoting the formation of a homogeneous polymer network. This compatibility enhances crosslinking density and ultimately improves the mechanical properties of the final adhesive.

Table 9 presents the costs of the chemical components used in the adhesive formulations. Substituting 45% of the PF resin with hydroxymethylated lignin results in a reduction of approximately 30% in the overall adhesive cost.

Comparing these results with those published in scientific articles is challenging. Although research on the manufacture of adhesives from lignin is extensive, the variety of tested possibilities makes it difficult to reach a consensus on a maximum substitution percentage, though it is generally agreed that better results are obtained when lignin is used in adhesive formulations. The results obtained are highly dependent on the wood source of the lignin, the isolation method used, and the adhesive manufacturing strategy. Most studies prepare adhesives where lignin is used in the resin manufacturing process, which was not the approach in this work. Regarding the type of lignin used, although results are reported for both kraft and soda lignins, most studies focus on coniferous lignins.

A summary of results with kraft lignin is provided, though there are also studies supporting the use of organosolv, soda lignins, and lignosulfonates in adhesive manufacturing. Ghorbani et al. [8] studied the production of boards with lignin–phenol–formaldehyde (LPF) resin-based adhesives, substituting 20% to 40% of phenol with various technical lignins (organosolv, soda, kraft, and lignosulfonates). They found that kraft pine lignin and lignosulfonates were the most suitable lignins for phenol replacement and noted no differences in the maximum breaking strength of panels when using methoxylated and non-methoxylated lignin, though higher pressing temperatures were required to cure the new adhesive [8,32,33]. Other studies by the same lead researcher found lower strengths with LPF resin compared to phenol–formaldehyde resins for a 40% substitution percentage [32].

In the study conducted by Solt et al. [34], an adhesive based on lignin–phenol–formaldehyde resin with 50% phenol substitution by unmodified pine kraft lignin was tested. The adhesives were tested on beech wood samples similar to those used in this work. The maximum strength recorded was lower for the LPF resin adhesive compared to the PF resin adhesive, with longer pressing times required for the former.

Abdelwahab and Nassar [35] produced LPF resins with up to 90% phenol substitution by unmodified bagasse kraft lignin. The results showed that the maximum strengths obtained with LPF-based adhesives were higher than with PF adhesives, even at high substitution percentages. This is explained by the fact that bagasse lignin is much more reactive than coniferous and hardwood kraft lignin.

In Xian et al. [36], coniferous kraft lignin was treated with a deep eutectic solvent (DES) composed of zinc chloride and lactic acid to produce lignin–phenol–formaldehyde resins with up to 70% phenol substitution. Plywood panels were made, achieving significantly higher maximum strengths than those obtained with phenol–formaldehyde resins. However, the viscosity of the LPF adhesive was substantially higher than that of PF adhesives, which could cause application issues.

Galdino et al. [37] produced adhesives from *Eucalyptus* kraft lignin, which was phenolated before manufacturing lignin–phenol–formaldehyde resins with substitution percentages of 10%, 20%, 30%, and 50%. The adhesives were used to make samples with pine wood, similar to the procedure in this work. They concluded that the adhesive prepared with 30% lignin substitution achieved similar results to the resin, but the adhesive with 50% substitution was not suitable for use due to performance issues.

In Rodrigues et al. [38], *Eucalyptus* kraft lignin and its soluble and insoluble fractions after ethyl acetate treatment were used to obtain low molecular weight, low dispersion lignin with high hydroxyl content. LPF resins were made by substituting 25% and 50% of phenol with these lignins. Adhesion was tested using birch samples on an Automated Bonding Evaluation System (ABES). They also tested lignin as an extender, similar to this work, by mixing commercial PF resin with lignin at a 15% substitution level. They concluded that for LPF resins, only a 25% phenol substitution with ethyl acetate-solubilized lignin produced results similar to the commercial resin, noting that this process is costly and complex. In contrast, using lignin as an extender produced similar results to commercial phenolic resin when using ethyl acetate-solubilized lignin, while unmodified lignin reduced adhesion strength.

In de Freitas et al. [39], *Eucalyptus* kraft lignin from “Suzano Papel e Celulose” was demethylated with hydrochloric acid and then subjected to basic phenolysis. Lignin-formaldehyde resins were prepared without additional phenol. Although phenol substitution percentages are not calculated in this study, the process could be compared to a 45% substitution, as phenolysis was performed with this proportion. The performance of the adhesives was tested using eucalyptus wood samples. Results showed that unmodified lignin had similar strength to the commercial adhesive, while demethylated lignins presented higher maximum breaking strength.

In de Magalhães et al. [40], lignin precipitated from black liquor obtained from a Brazilian pulp mill processing *Eucalyptus* was treated thermally at 300 °C for 6 min and oxidized with potassium dichromate at 40 °C for 2 h. Resins were made with 50% phenol substitution for both unmodified and modified lignins. Three-layer plywood panels were made, with no significant differences in shear strength compared to phenol–formaldehyde adhesives.

The bibliographic results previously reported by different researchers cannot be directly compared with those found in this study, as they involve the use of different types of lignin (from conifers or bagasse), or, in the case of *Eucalyptus* lignin, it is used to manufacture the resin (LPF resin) rather than as an extender. Nevertheless, these results are presented here because the substitution percentages achieved are of the same magnitude as those used in this work. Using *Eucalyptus* lignin as an extender, rather than for the production of LPF resin, results in a much simpler process for adhesive manufacture. This approach can be directly implemented in mills producing engineered wood products and provides a viable option for many companies that import PF resin without the ability to modify it.

## 4. Conclusions

The experimental results allow us to conclude that it is feasible to formulate phenol–formaldehyde adhesives by partially substituting the base resin with lignin as an extender. Activating the lignin enhances the substitution percentage, which reduces the final cost of the adhesive.

Among the selected activation methods, hydroxymethylation yields statistically superior results in terms of maximum tensile strength of the test specimens, while the remaining methods do not significantly affect the strength. Therefore, it is expected that using hydroxymethylated lignin may achieve higher substitution percentages than with unmodified lignin.

Regarding the activation of lignin through acid or alkaline phenolysis, although this method has shown promising results in the literature for the production of lignin–phenol–formaldehyde resins, the findings of this study suggest that it may not be a viable alternative when lignin is used as an extender in wood adhesive formulations.

The method of incorporating hydroxymethylated lignin as an extender during adhesive manufacturing, rather than using it in resin production, has proven to be an efficient strategy for partially substituting phenol–formaldehyde resin with lignin, achieving results comparable to those reported in the literature.

In conclusion, it is essential to clarify that the primary objective of this study was to evaluate the feasibility of formulating adhesives using modified lignin and to assess the strength of the resulting formulations. This work should be further complemented by studies that apply these adhesives in the production of specific products, such as plywood, to determine whether superior performance can be achieved with the lignin-modified adhesive. Additionally, future studies could investigate whether the enhanced adhesive strength allows for a reduction in the amount of adhesive used, thereby optimizing material efficiency. Further tests should also be conducted to evaluate adhesion properties, including water resistance, aging resistance, and storage stability.

## Figures and Tables

**Table 1:** Formulation of Base Adhesive and Adhesives Modified with Hydroxymethylated Lignin.

Adhesive	0% (Base)	10%	15%	20%	30%	45%	60%
PF Resin (43%) (% *w*/*w*)	66.9	60.2	56.9	53.5	46.8	36.8	25.7
Hydroxymethylated Lignin (27%) (% *w*/*w*)	0	10.6	16.0	21.3	32.0	47.9	61.4
Walnut Flour (% *w*/*w*)	5.7	5.7	5.7	5.7	5.7	5.7	5.7
Wheat Flour (% *w*/*w*)	7.6	7.6	7.6	7.6	7.6	7.6	7.3
Soda (50%) (% *w*/*w*)	3.1	2.6	2.4	2.1	1.7	1.0	0
Water (% *w*/*w*)	16.7	13.3	11.4	9.8	6.2	1.0	0
Viscosity (cP)	2000–4500						

**Table 2:** Formulation of Adhesives with Unmodified Lignin and Lignin Modified by Acidic and Basic Phenolysis.

Adhesive	0% (Base)	10%	15%	20%	30%	45%	60%
PF Resin (43%) (% *w*/*w*)	66.9	60.2	56.8	53.6	46.8	36.8	26.7
Solid Lignin (% *w*/*w*)	0	2.9	4.3	5.8	8.6	13.0	17.4
Walnut Flour (% *w*/*w*)	5.7	5.7	5.7	5.7	5.7	5.7	5.7
Wheat Flour (% *w*/*w*)	7.6	7.6	7.6	7.6	7.6	7.6	7.6
Soda (50%) (% *w*/*w*)	3.1	3.1	3.1	3.1	3.1	3.1	3.1
Water (% *w*/*w*)	16.7	20.5	22.5	24.2	28.2	33.8	39.5
Viscosity (cP)	2000–4500						

**Table 3:** Characterization of raw lignins by phenolic OH content, S/G ratio and molecular weight distribution (Mn, Mw and PDI). The errors indicated correspond to the standard deviation.

Starting Lignin	Phen OH (mmol/g)	S/G Ratio	Mn (Da)	Mw (Da)	PDI
KL1	5.5 ± 0.1	3.1 ± 0.5	1015 ± 25	2790 ± 185	2.7 ± 0.1
KL2	5.3 ± 0.2	3.0 ± 0.4	1682 ± 38	3090 ± 150	1.8 ± 0.2
SL1	1.8 ± 0.2	3.0 ± 0.4	1581 ± 43	7906 ± 125	5.0 ± 0.3
SL2	2.1 ± 0.1	2.9 ± 0.3	1506 ± 45	7875 ± 120	5.2 ± 0.3
SL3	2.1 ± 0.1	2.8 ± 0.3	2321 ± 23	8269 ± 159	3.6 ± 0.2

**Table 4:** ANOVA Comparison of the Base Adhesive with Adhesives Formulated with kraft lignin precipitated with CO_2_.

Adhesive	τ_m_ (N/mm^2^)	Standard Error	Homogeneous Groups	Significant Difference with Base Adhesive
REFERENCE	9.10	0.16	abc	-
KL1_UM_10	8.18	0.98	abc	No
KL1_UM_20	8.89	1.17	abc	No
KL1_UM_30	7.00	0.55	ab	No
KL1_HM_10	9.60	0.27	bc	No
KL1_HM_20	10.70	1.09	c	No
KL1_HM_30	8.93	0.26	abc	No
KL1_BP_10	8.45	0.90	abc	No
KL1_BP_20	8.99	1.16	abc	No
KL1_BP_30	6.42	1.11	a	No
KL1_AP_10	9.11	1.27	abc	No
KL1_AP_20	7.09	0.57	ab	No
KL1_AP_30	7.29	1.61	ab	No

**Table 5:** ANOVA Comparison of the Base Adhesive with Adhesives Formulated with kraft ligning precipitated with H_2_SO_4_.

Adhesive	τ_m_ (N/mm^2^)	Standard Error	Homogeneous Groups	Significant Difference with Base Adhesive
REFERENCE	9.10	0.16	bcd	-
KL2_UM_10	8.22	0.65	abcd	No
KL2_UM_20	9.28	0.81	cde	No
KL2_UM_30	7.38	0.26	ab	No
KL2_HM_10	9.67	0.44	de	No
KL2_HM_20	10.73	0.32	e	Yes
KL2_HM_30	9.70	0.61	de	No
KL2_BP_10	7.82	0.50	abc	No
KL2_BP_20	8.74	0.74	bcd	No
KL2_BP_30	7.88	0.76	abc	No
KL2_AP_10	8.73	0.60	bcd	No
KL2_AP_20	7.36	1.01	ab	No
KL2_AP_30	6.91	0.28	a	Yes

**Table 6:** ANOVA Comparison of the Base Adhesive with Adhesives Formulated with soda lignin precipitated with HCl.

Adhesive	τm (N/mm^2^)	Standard Error	Homogeneous Groups	Significant Difference with Base Adhesive
REFERENCE	9.10	0.16	cd	-
SL1_UM_10	8.22	0.65	abcd	No
SL1_UM_20	9.28	0.81	cd	No
SL1_UM_30	7.38	0.26	abc	No
SL1_HM_10	10.51	0.35	d	No
SL1_HM_20	13.72	1.18	e	Yes
SL1_HM_30	13.59	1.32	e	Yes
SL1_BP_10	7.89	1.03	abcd	No
SL1_BP_20	5.52	0.89	a	Yes
SL1_BP_30	5.89	0.67	ab	Yes
SL1_AP_10	8.63	0.74	bcd	No
SL1_AP_20	7.69	2.05	abcd	No
SL1_AP_30	8.38	1.66	abcd	No

**Table 7:** ANOVA Comparison of the Base Adhesive with Adhesives Formulated with soda lignin precipitated with H_2_SO_4_.

Adhesive	τm (N/mm^2^)	Standard Error	Homogeneous Groups	Significant Difference with Base Adhesive
REFERENCE	9.10	0.16	cde	-
SL2_UM_10	8.22	0.65	abcd	No
SL2_UM_20	9.28	0.81	de	No
SL2_UM_30	7.38	0.26	abcd	No
SL2_HM_10	10.32	0.76	e	No
SL2_HM_20	13.50	0.97	f	Yes
SL2_HM_30	12.70	1.11	f	Yes
SL2_BP_10	7.07	0.41	ab	Yes
SL2_BP_20	7.66	0.68	abcd	No
SL2_BP_30	8.59	0.80	bcde	No
SL2_AP_10	6.43	0.32	a	Yes
SL2_AP_20	7.21	0.47	abc	No
SL2_AP_30	6.34	0.72	a	Yes

**Table 8:** ANOVA Results of tensile shear strength for Adhesives Processed with KL2 and SL2.

Adhesive	τ_m_ (N/mm^2^)	Standard Error	Homogeneous Groups	Significant Difference with Reference Adhesive
REFERENCE	14.19	1.13	abcd	-
KL2_UM_15	14.17	1.69	abcde	no
KL2_UM_30	16.92	1.15	defgh	no
KL2_UM_45	13.49	1.31	abc	no
KL2_HM_15	19.15	0.57	fgh	yes
KL2_HM_30	20.79	0.65	gh	yes
KL2_HM_45	18.08	0.55	efgh	yes
KL2_BP_15	15.50	1.89	abcdef	no
KL2_BP_30	16.23	2.17	bcdefg	no
KL2_BP_45	11.84	0.63	ab	no
KL2_AP_15	15.81	1.05	bcdef	no
KL2_AP_30	15.57	1.54	bcdef	no
KL2_AP_45	15.08	0.94	abcdef	no
SL2_UM_15	13.46	2.13	abc	no
SL2_UM_30	12.70	1.10	abc	no
SL2_UM_45	10.96	1.87	a	no
SL2_HM_15	18.75	2.42	fgh	yes
SL2_HM_30	21.22	1.75	h	yes
SL2_HM_45	20.89	1.24	h	yes
SL2_BP_15	12.42	1.86	abc	no
SL2_BP_30	12.89	2.01	abc	no
SL2_BP_45	12.72	1.73	abc	no
SL2_AP_15	16.86	1.65	cdefgh	no
SL2_AP_30	19.60	2.26	fgh	Yes
SL2_AP_45	18.22	2.40	fgh	yes

**Table 9:** Cost of adhesive chemicals.

Component	Cost (USD/Ton)
PF Resin (43%)	1400
Kraft or soda lignin	500
NaOH (solution 50%)	425
Formaldehyde (solution 27%)	345
Walnut flour (%)	50
Wheat flour (%)	70

## Data Availability

The original contributions presented in this study are included in the article. Further inquiries can be directed to the corresponding author.

## Abbreviations

The following abbreviations are used in this manuscript for lignin identification:

KL (kraft lignin), SL (soda lignin), UM (unmodified lignin), HM (hydroxymethylation), AP (acid phenolysis), BP (basic phenolysis), Mn (number average molecular weight), Mw (weight average molecular weight), PDI (polydispersity index = Mn/Mw).
